# Modulation of host immune defenses by *Aeromonas* and *Yersinia* species: convergence on toxins secreted by various secretion systems

**DOI:** 10.3389/fcimb.2013.00070

**Published:** 2013-10-30

**Authors:** Jason A. Rosenzweig, Ashok K. Chopra

**Affiliations:** ^1^Department of Biology, Center for Bionanotechnology and Environmental Research, Texas Southern UniversityHouston, TX, USA; ^2^Department of Environmental and Interdisciplinary Sciences, Texas Southern UniversityHouston, TX, USA; ^3^Department of Microbiology and Immunology, University of Texas Medical BranchGalveston, TX, USA; ^4^Sealy Center for Vaccine Development, University of Texas Medical BranchGalveston, TX, USA; ^5^Institute of Human Infections and Immunity, University of Texas Medical BranchGalveston, TX, USA; ^6^Galveston National Laboratory, University of Texas Medical BranchGalveston, TX, USA

**Keywords:** type 2-, -3, and -6 secretion systems, apoptosis, pyroptosis, actin cytoskeleton, effector proteins

## Abstract

Like other pathogenic bacteria, *Yersinia* and *Aeromonas* species have been continuously co-evolving with their respective hosts. Although the former is a bonafide human pathogen, the latter has gained notararity as an emerging disease-causing agent. In response to immune cell challenges, bacterial pathogens have developed diverse mechanism(s) enabling their survival, and, at times, dominance over various host immune defense systems. The bacterial type three secretion system (T3SS) is evolutionarily derived from flagellar subunits and serves as a vehicle by which microbes can directly inject/translocate anti-host factors/effector proteins into targeted host immune cells. A large number of Gram-negative bacterial pathogens possess a T3SS empowering them to disrupt host cell signaling, actin cytoskeleton re-arrangements, and even to induce host-cell apoptotic and pyroptotic pathways. All pathogenic yersiniae and most *Aeromonas* species possess a T3SS, but they also possess T2- and T6-secreted toxins/effector proteins. This review will focus on the mechanisms by which the T3SS effectors *Yersinia* outer membrane protein J (YopJ) and an *Aeromonas hydrophila* AexU protein, isolated from the diarrheal isolate SSU, mollify host immune system defenses. Additionally, the mechanisms that are associated with host cell apoptosis/pyroptosis by *Aeromonas* T2SS secreted Act, a cytotoxic enterotoxin, and Hemolysin co-regulated protein (Hcp), an *A. hydrophila* T6SS effector, will also be discussed.

## Introduction

Intricate host-pathogen interactions are constantly evolving as the latter has to combat formidable host immune defenses, resulting in expression/de-repression of genes and molecular mimicry. In some instances, strong immune responses to microbes select for escape mutants with the latter further honing the immune response to that variant pathogen, leading to an ongoing battle, as can be seen typically in human immunodeficiency virus escape mutants (Akahoshi et al., 2012; Yagita et al., 2013) and in *Chlamydia trachomatis* (Nunes et al., 2010). One classic example of this paradigm is programmed host cell death (caused by apoptosis, pyroptosis, and necrosis) which could benefit the host immune system (if clearing an intracellular pathogen) or could be co-opted by the pathogen as a means of eliminating undesirable host cells (e.g., innate immune cells) (Ulett and Adderson, 2006).

Whereas apoptosis is a “self-contained” event that does not stimulate a robust inflammatory response, both pyroptosis and necrosis of host cells release pro-inflammatory cytokines and their cytoplasmic contents into the extra-cellular milieu (Lamkanfi and Dixit, 2010). Apoptosis is a caspase-dependent process that drives embryonic development and is largely characterized by nuclear fragmentation and condensation, blebbing of the plasma membrane, and cell shrinkage. Since all of these physiological consequences occur intracellularly, no host cell cytoplasmic content is released into the extracellular environment, thereby preventing inflammation (Strasser et al., 2000).

Necrosis, by contrast, is a caspase-independent process that results in host cell swelling, disorganized and extensive chromatin hydrolysis, and cytoplasmic leakage (Berghe et al., 2010). Finally, caspase-1-dependent pyroptosis leads to secretion of pro-inflammatory cytokines interleukin-1β (IL-1β) and IL-18. Caspase-1, interestingly, is not involved in apoptosis and is activated by one of four inflamasomes, which contain a member of the nucleotide-binding oligomerization domain-containing protein (Nod)-like receptor family, during pyroptosis (Lamkanfi and Dixit, 2009). Surprisingly, the pathogenic yersiniae can induce apoptosis, necrosis, and pyroptosis depending on the host-cell type infected (Monack et al., 1997; Ruckdeschel et al., 1997, 1998, 2001; Bergsbaken and Cookson, 2007; Zheng et al., 2012). Similarly, *Aeromonas* species are also able to induce apoptosis (through various caspase activation) (Galindo et al., 2004b, 2006a,b; Martins et al., 2007; Su et al., 2007; Sierra et al., 2007, 2010).

Gram-positive pathogens have also been shown to induce apoptosis in various cell types by disparate mechanisms. Non-secreted lipotechoic acids (LTAs), well-conserved surface antigens on a wide variety of Gram-positive organisms, are such examples that induce apoptosis by distinct mechanisms in various cell types (Ulett and Adderson, 2006). Beyond LTAs that induce host cell apoptosis, *Bacillus anthracis* employs its lethal factor exotoxin (Park et al., 2002; Popov et al., 2002), *Listeria monocytogenes* utilizes listeriolysin, a cytolysin (Carrero et al., 2004), while the streptococci employ hemolysins (Ring et al., 2002; Liu et al., 2004). Ultimately, Gram-positive pathogens' apoptosis-inducing mechanisms are very diverse; they can typically be either intrinsic (e.g., mitochondrial dysfunction) or extrinsic whereby death domains are activated (Ulett and Adderson, 2006). Of the 27-members that belong to the *Aeromonadaceae* family, *A. hydrophila*, *A. veronii*, and *A. caviae* are frequently isolated as human pathogens, with most infections contracted *via* the fecal-oral route or through wounds (Altwegg et al., 1991; Kirov, 1993; Palu et al., 2006; Edberg et al., 2007). *Aeromonas hydrophila* is also a fish pathogen that can negatively impact the fishing industry. The majority of human *Aeromonas* infections result in self-limiting gastroenteritis or superficial skin infections. However, more threatening systemic infections include bacteremia, cellulitis, peritonitis, hemolytic-uremic syndrome, and necrotizing fasciitis (Chopra et al., 1993, 1996; Janda et al., 1994; Merino et al., 1995; Kuhn et al., 1997; Chopra and Houston, 1999; Minnaganti et al., 2000; Brouqui and Raoult, 2001; Galindo et al., 2006a,b; Sha et al., 2013). Alarmingly, it seems as though instances of *Aeromonas*-induced necrotizing fasciitis are on the rise (Huang et al., 2011; Chang et al., 2012; Kao and Kao, 2012; Wu et al., 2012).

Of the 11 known Gram-negative *Yersinia* species, only *Y. enterocolitica*, *Y. pseudotuberculosis*, and *Y. pestis* are human pathogens. Strikingly, while *Y. pseudotuberculosis* and *Y. enetrocolitica* cause self-limiting gastroenteritis (Galindo et al., 2011), *Y. pestis* (transmitted by the bite of an infected flea) causes radically different diseases (bubonic, septicemic, or pneumonic plague) which have resulted in three major human pandemics as well as the great plagues of London in the mid-late 1600s (Inglesby et al., 2000). Currently, the plague-causing bacterium can be treated with various antibiotics (Rosenzweig et al., 2011a), with levofloxacin recently being approved by the Food and Drug Administration against all forms of plague. However, there is no vaccine against this deadly pathogen (Rosenzweig et al., 2011b; Rosenzweig and Chopra, 2012).

As earlier mentioned, representatives of both the *Yersinia* and *Aeromonas* species are capable of causing gastroenteritis following the fecal-oral route of infection, and they similarly possess a type three secretion system (T3SS). The T3SS multiprotein complex/hyperstructure (Norris et al., 2012) is evolutionarily related to the bacterial flagella (Nguyen et al., 2000; Gophna et al., 2003) and enables rapid translocation of effector proteins directly into the targeted host cell cytoplasm, resulting in a number of anti-host consequences. Interestingly, whereas the yersiniae possess well-studied T3SS weaponry, *Aeromonas* species harbor well-defined T3- and T6-secretion systems (derived from phage injection machinery) along with its two identified effector protein substrates, Hemolysin co-regulated proteins (Hcps) and Valine glycine repeat G proteins (VgrGs) (Sierra et al., 2007, 2010; Vilches et al., 2009; Bergh et al., 2013; Sha et al., 2013). Interestingly, hemolysins in *Streptococcus agalactiae*, a Group B streptococci, have also been shown to induce apoptosis in phagocytic cells (Ulett and Adderson, 2006 and references therein). The yersiniae T6SSs have not been as extensively characterized; however, in *Y. psuedotuberculosis*, it is regulated by the transcriptional factor OmpR and appears to play a role in stress responses, quorum sensing, and maintenance of internal pH homeostasis (Zhang et al., 2011, 2013; Gueguen et al., 2013). In *Y. pestis*, the T6SS was found to secrete an Hcp-like autoagglutination factor (Podladchikova et al., 2011). Finally, *Aeromonas* species also employ the general secretory T2SS pathway to export cytotoxic enetrotoxin Act (with hemolytic, cytotoxic, and enterotoxic activities) into the extracellular milieu (Chopra and Houston, 1999). Within the yersiniae, the T3SS injects into the host 7 *Yersinia* outer membrane protein (Yop) effector proteins that have been identified as YopP/J, -H, -E, YopO/YpkA, YopT, YopK, and YopM; these Yops counteract host immune defenses by various mechanisms (Viboud and Bliska, 2005). Upon first encountering a Gram-negative pathogen, like *Y. pestis*, innate immune cells (e.g., macrophages and/or dendritic cells) recognize non-specific, pathogen-associated molecular patterns/microbe-associated molecular patterns (PAMPs/MAMPs), like lipopolysaccharide (LPS), lipoprotein, or flagellin. When PAMPs/MAMPs associate with their recognition receptors, e.g., Toll-like receptors (TLRs), various mitogen-activated protein (MAP) kinase (MAPK) and nuclear factor Kappa B (NF-κB) signaling pathways are activated resulting in the upregulation of IL-12, -18, and tumor necrosis factor alpha (TNF-α) proinflammatory cytokine production (Matsumoto and Young, 2009).

The yersiniae counteract the aforementioned inflammatory response when YopP/J acetylate I kappa B kinase (IKK) and MAPK kinases (MKKs), preventing their phosphorylation and subsequent activation. The disruption in these signaling events results in innate immune cells undergoing apoptosis (Orth, 2002; Mittal et al., 2006; Mukherjee et al., 2006). A more detailed description of *Yersinia* outer membrane protein J (YopJ) mechanisms of mollifying host defenses is discussed in a later section. YopE, -H, -T, and YopO/YpkA all operate to disrupt actin cytoskeleton re-arrangements and phagocytosis, albeit by attacking unique and distinct targets. YopE is a GTPase-activating protein (GAP), while YopT targets Rac-1, RhoA and Cdc-42, and YopH, which is a tyrosine phosphotase, primarily targets focal adhesion complexes. YopO/YpkA, through its kinase activity, also targets Rac-1 and RhoA as well as actin directly. YopM localizes to the target cells' nuclei and disrupts cytokine IL-15 production by targeting ribosomal S6 protein kinase 1 (RSK1) and possibly protein kinase C-like 2 (PRK2) (Matsumoto and Young, 2009 and references therein). Finally, YopK was found to associate with the translocation pore and is believed to modulate inflammation (Brodsky et al., 2010).

As mentioned earlier, many *Aeromonas* species also possess a T3SS. In fact, within the fish pathogen *A. salmonicida*, four T3SS-associated effectors have been identified: AexT, AopP, AopH, and AopO (Braun et al., 2002; Dacanay et al., 2006; Fehr et al., 2006). Our laboratory recently identified an AexT-like protein (a novel T3SS effector, AexU) in a diarrheal isolate SSU of *A. hydrophila* (Sha et al., 2007). While *Aeromonas* outer protein P (AopP) disrupts NF-κB signaling downstream of IKKB, unlike YopJ in the yersinaie, it does not disrupt the MAPK signaling pathway (Fehr et al., 2006). On the contrary, AexT and AexU both possess highly cytotoxic ADP-ribosyltranferase activity for host proteins (Braun et al., 2002; Sha et al., 2007). AopO and AopH remain poorly understood and are homologues of yersiniae YopO/YpkA and YopH, respectively (Sha et al., 2007). Interestingly, we also demonstrated that an *A. hydrophila* Δ*aopB* deletion mutant, unable to translocate effector Aops into host cells, exhibited greatly reduced virulence in a murine model of infection (Sha et al., 2005).

### T3SS effector YopJ's mechanisms of anti-host activity

The yersiniae T3SS effector YopJ is an acetyltransferase as well as a de-ubiquitinase. Its anti-host activity involves blocking MAPK signaling and NF-κB activation (Table 1). This aberrant signaling leads to significantly reduced production of both proinflammatory and anti-apoptotic host cytokines (Monack et al., 1997; Orth et al., 1999, 2000; Mukherjee et al., 2006). Shedding more light on the mechanism of YopJ anti-host activity, a report from Shrestha et al. (2012) identified that YopJ reduced the induction of eukaryotic initiation factor 2 (eIF2) in both yeast and mammalian cells, and that eIF2 signaling was required for YopJ-mediated inhibition of NF-κB activation as well as pro-inflammatory cytokine production.

**Table 1:** **The mechanisms of action of yersiniae effectors YopJ and YopK**.

**Effector**	**Secretion system**	**Mechanisms of pathogenesis**	**References**
YopJ	T3SS	Acetyltransferase and deubiquitinase that blocks MAPK and NF-κB signaling, causing reduced production of pro-inflammatory and anti-apoptotic cytokines.	Monack et al., 1997; Orth et al., 1999, 2000; Mukherjee et al., 2006
		Reduced induction of eukaryotic initiation factor 4.	Shrestha et al., 2012
		Signals through TLR-2 to increase production of Caspase-3, -8, IRAK-4, FADD.	Pandey and Sodhi, 2011
		Serine threonine acetylation of TAK1 in *Drosophila* preventing its phosphorylation.	Paquette et al., 2012
		Blocks interaction of RICK and Nod2 acetylation of RICK and TAK 1; Nod2 then interacts with caspase-1 to increase expression of IL1-β which promotes bacterial dissemination through the gut.	Meinzer et al., 2012
YopK	T3SS	Regulates pyroptosis (via caspase-1).	Brodsky et al., 2010
		Regulates YopJ-mediated apopotosis in macrophages and facilitates bacterial dissemination.	Peters et al., 2013

*MAPK, mitogen-activated protein kinases; NF-κB, nuclear factor Kappa B; TLR-2, toll-like receptor 2; IRAK 4, interleukin 1 receptor associated kinase; FADD, Fas-associated death domain; TAK1, tumor growth factor β-activated kinase; RICK, receptor interacting protein 1-like interacting caspase-like apopotosis regulatory protein kinase; Nod2, nucleotide-binding oligomerization domain-containing protein 2; IL 1-β, interleukin 1 Beta; T3SS, type three secretion system; Yop, Yersinia outer protein*.

#### TLR-2, NF-κB, and Nod2 signaling pathways all targeted by the versatile YopJ

By using recombinant YopJ (rYopJ), it was determined that TLR-2 in murine macrophages was involved in YopJ-mediated apoptotic signaling by increasing production of caspases 3 and 8, IL-1 receptor associated kinase (IRAK)-4, Fas-associated protein with death domain (FADD), and phosphorylation of IκB and MAPK (Pandey and Sodhi, 2011). Together with this TLR-2 apoptotic signaling, the ability of YopJ to target macrophage eIF2 signaling pathway required for inhibition of NF-κB activation as well as pro-inflammatory cytokine production ultimately leads to host cell apoptosis (Shrestha et al., 2012). In a separate study employing a *Drosophila* model system, transforming growth factor (TGF)-β-activated kinase (TAK1), which is part of the immune NF-κB signaling pathway independent of the TLR-2 signaling, was identified as the YopJ serine/threonine acetylation target (Paquette et al., 2012). Following YopJ acetylation of serine/threonine residues in the active site of *Drosophila* TAK1, its phosphorylation was blocked preventing activation of this kinase. Corroborating *Drosophila* studies, YopJ similarly modified and inhibited TAK1 in mammalian cells (Paquette et al., 2012).

Despite an earlier study demonstrating rYopJ activation of TLR-2 signaling in macrophages (Pandey and Sodhi, 2011), *in vivo* studies employing both *Drosophila* and macrophage models of infection clearly demonstrated that native YopJ indeed activated the NF-κB signaling but not the TLR-2 signaling pathway (Paquette et al., 2012). It was proposed that following acetylation of key serine/threonine residues in the active sites of both RIP (receptor interacting protein 1)-like interacting caspase-like apoptosis regulatory protein kinase (RICK) and TAK1, YopJ prevented the interaction of RICK and Nod2, a NACHT-leucine-rich repeats (NLRs) recognition receptors. Further, Nod2 interacted with caspase 1, promoting increased expression/production of IL1-β and dissemination of *Y. psudeotuberculosis* through the gut epithelium (Meinzer et al., 2012). Perhaps this seeming contradiction of YopJ not signaling through TLR-2 during *in vivo* studies while rYopJ was shown to signal through TLR-2 *in vitro* underscores one very important point. Different cell types and/or organisms likely possess disparate/specialized receptors used to recognize threatening pathogens. Viewed in this light, one can envision how within one cell type there could exist several unique receptors that could detect the same (or different products) derived from one pathogen. In the very delicate host-pathogen paradigm, every potential detection mechanism must be employed if the host is to successfully subvert the pathogenic threat. Collectively, YopJ has been observed disrupting TLR-2, Nod2, and the NF-κB signaling pathways, making the T3SS-acetyltransferase a potent weapon for the pathogenic yersiniae.

#### The yopK “switch” for YopJ activity

Importantly, YopJ seems to function in concert with another Yop, YopK, which regulates YopJ activity (Table 1). Studies have shown that YopK appeared to regulate pyroptosis (via caspase 1) by *Y. pseudotuberculosis* (Brodsky et al., 2010) and YopJ-dependent apoptosis specifically in RAW 264.7 monocytic cells, thereby facilitating bacterial dissemination in a murine model of pneumonic plague (Peters et al., 2013). Despite YopK appearing dispensable for *Y. pseudotuberculosis* to induce caspase-1 mediated pyroptosis (Brodsky et al., 2010), it was required for optimum virulence of *Y. pestis* in a pneumonic murine model of infection (Peters et al., 2013). Further, cell culture *Y. pestis* infection models have also revealed that caspase 1 activation occurs downstream of cell necrosis, is independent of mitochondrial driven apoptosis, but does require cathepsin B activity (Zheng et al., 2012). Taken together, these data demonstrated that, at least in *Y. pestis*, YopJ's ability to induce apoptosis is regulated by a YopK “switch” downstream of cell necrosis.

### T3SS effector AexU's mechanisms of anti-host activity

Shortly after a T3SS was identified in both *A. salmonicida* and *A. hydrophila* and implicated in their pathogenesis of both fish and animal/human hosts (Burr et al., 2002; Yu et al., 2004; Sha et al., 2005), we identified a novel T3SS-dependent AexT-like protein, referred to as AexU, in *A. hydrophila* (Sha et al., 2007; Sierra et al., 2007). AexT (Table 2), identified in the fish pathogen *A. salmonicida*, is homologous to the *Pseudomonas aeruginosa* ExoT/S and is also a bifunctional effector (Pederson et al., 1999; Sundin et al., 2004). Its amino terminus has YopE-like activity of yersiniae and can depolymerize actin by targeting RhoA, while its carboxy-terminus has highly cytotoxic ADP-ribosyltransferase (ADP-RT) activity for host proteins (Braun et al., 2002).

**Table 2:** **Aeromonad effector proteins AexT, AexU, Act, and Hcp mechanisms of action**.

**Effector**	**Secretion system**	**Mechanism of pathogenesis**	**References**
AexT	T3SS	Amino terminal activity targets RhoA and promotes actin depolymerization; carboxy terminal ADP-ribosyltransferase activity.	Braun et al., 2002
AexU	T3SS	Bifunctional-like AexT; activation of caspase-3 and -9 and induction of cell rounding, chromatin condensation; also required for virulence in mice.	Sierra et al., 2007
		GAP-activity (amino terminus) promotes apoptosis and disrupts the cell cytoskeleton as well as NF-κB signaling; prevents signaling of c-Jun, JNK, IκBα, and inhibits IL-6 and IL-8 secretion.	Sierra et al., 2010
		GAP-activity disruption of actin cytoskeleton mediated by down-regulating Rac-1; binding to β4-integrin results in cytotoxicity.	Abolghait et al., 2011
Act 2	T2SS	Induced upregulation of apoptosis-related genes.	Galindo et al., 2003
		Activates MEK1, JNK, ERK1/2, and c-Jun of the MAPK pathway; induces membrane blebbing and increased production of mitochondrial cytochrome C, caspase-3, -8, and -9.	Galindo et al., 2004b
Hcp	T6SS	Caspase 3 activation.	Suarez et al., 2008
		Demonstrates anti-phagocytic properties.	Suarez et al., 2010
		Hcp-2 is part of the T6SS apparatus while Hcp-1 negatively regulates motility and protease production.	Sha et al., 2013

*RhoA, Rat sarcoma homolog gene family member A; GAP, GTPase activating protein; NF-κB, nuclear factor Kappa B; JNK, c-Jun N-terminal kinase; IL, interleukin; Rac-1, Rac GTPase activating protein 1; MEK1, MAP/ERK kinase 1; ERK1/2, extracellular signal-regulated kinase 1/2; MAPK, mitogen-activated protein kinases*.

While the amino terminus of AexU from *A. hydrophila* SSU maintained ~67% sequence similarity to its AexT counterpart, surprisingly, the AexU carboxy terminus had a unique sequence which did not share similarity with any other known protein in the NCBI database, despite full-length AexU maintaining ADP-RT activity (Sha et al., 2007). Surprisingly, the purified full-length, truncated amino terminus, or truncated carboxy terminus of AexU all exhibited ADP-RT activity; however, the full-length AexU and its amino terminus exhibited higher ADP-RT activity than did the carboxy terminus of AexU alone (Sierra et al., 2007). Since the ADP-RT activity of the *Pseudomonas* homologue (ExoT/S) resides in its carboxy terminus, the aforementioned finding suggested a potentially unique evolution of AexU as an *Aeromonas* T3SS effector.

**Figure 1:**
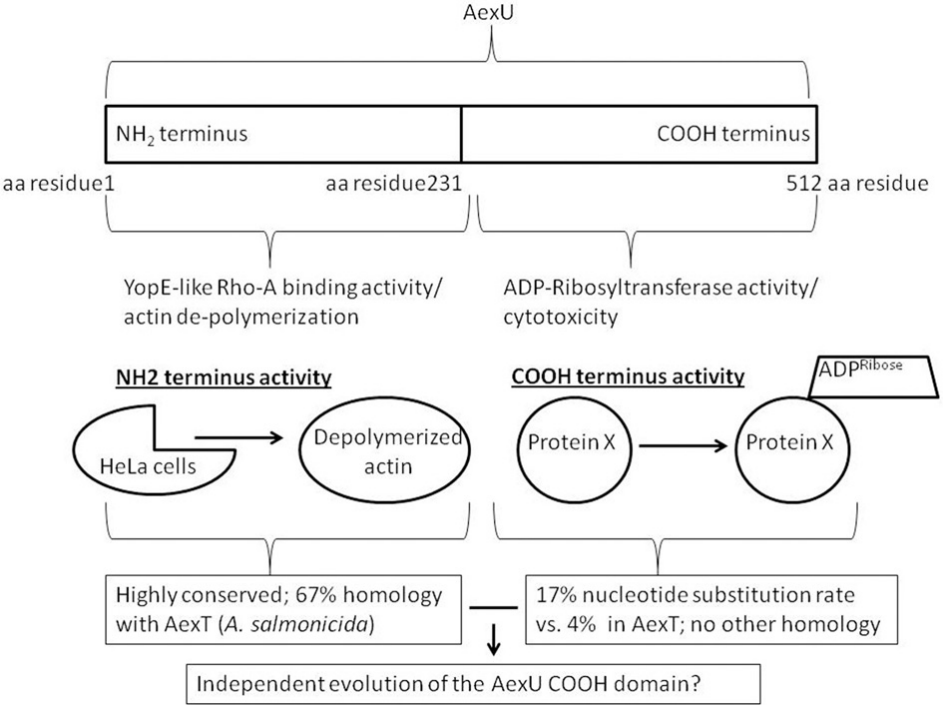
**The bifunctional AexU effector protein.** The two independent activities of AexU are localized on either the NH2- or the COOH-termini. This allows for the possibility of an independently evolving COOH-terminal activity.

Supporting this view, findings from a comprehensive genomic study evaluating an *A. veronii* group collection (derived from both clinical and environmental isolates) revealed that all 20 bacterial isolates possessed a functional T3SS as well as both AexU and AexT effectors. However, whereas AexU had a nucleotide substitution rate of ~17% in its carboxy terminal region, AexT was much better conserved and demonstrated only a ~4% substitution rate (Silver and Graf, 2009). Perhaps, the AexU carboxy terminus is evolving independently from its amino terminus allowing for not only the possibility of producing varying alleles but also producing a variety of AexU effectors capable of adapting to changing environments within the host (Figure 1)?

#### Immunogenicity of AexU and its contribution to overall virulence

In a cell culture infection model, our laboratory found that *A. hydrophila* AexU (Table 2) caused actin reorganization and cell rounding, chromatin condensation, and the activation of caspase 3 and 9, all hallmark features of apoptosis (Sierra et al., 2007). Furthermore, we reported that *A. hydrophila* AexU also possessed GAP activity which strongly promoted apoptosis and disrupted actin cytoskeletal rearrangements of the host cells (Sierra et al., 2010). Additionally, it was noted that *A. hydrophila* AexU prevented phosphorylation of c-Jun [a component of the activator protein 1 (AP-1) transcription factor], c-Jun N-terminal kinase (JNK) and IκBα (thereby disrupting their signaling cascades), and inhibited IL-6 and -8 secretion from HeLa cells. Ultimately, AexU inhibited NF-κB and inactivated Rho GTPases in the host cell (Sierra et al., 2010). For reasons unclear at this time, an AexU variant devoid of both GAP and ADP-RT activities, when produced from an *aexU* null mutant of *A. hydrophila*, induced higher mouse mortality and increased pro-inflammatory cytokine production (Sierra et al., 2010). As was noted with YopJ, perhaps, to increase overall bacterial virulence through inflammation, evolutionary deactivation of AexU's activities provides a valuable “switch” for responding to various hosts?

In *P. aeruginosa*, it has been shown that the maturation of IL-1β is negatively regulated by ExoS and is dependent on its ADP-RT activity. In other words, ExoS devoid of this enzymatic activity when produced from the bacteria led to increased IL-1β production and pyroptosis of the host cells. However, AexU seemed to behave differently compared to ExoS as the former without the enzymatic activities was unable to alter IL-1β levels (Sierra et al., 2010). *A. veronii* AexU, in a GAP-dependent manner, was able to similarly disrupt actin cytoskeleton by down-regulating Rac-1 in HeLa cells (Abolghait et al., 2011). Additionally, *A. veronii* AexU was found to co-localize with β4-integrin resulting in cytotxicity for the host cells (Abolghait et al., 2011). Collectively, these data strongly demonstrated AexU's versatility as an effector protein by virtue of its ability to disrupt cell signaling, paralyze the host cell, activate caspases (initiating apoptosis), and interact with β4-integren promoting host cell cytotoxicty. When evaluating AexU's contribution to mouse virulence and immunogenicity, we found that an *A. hydrophila* Δ*aexU* deletion mutant caused significantly less mortality (40% compared to 90–100%) in intraperitoneally-challenged mice than did infection with the isogenic parental strain (Sha et al., 2007). Importantly, rAexU provided protective immunity to mice when subsequently challenged with *A. hydrophila* (Sha et al., 2007). Additionally, the *A. hydrophila* Δ*aexU* deletion mutant was unable to disseminate within infected mice leaving their lungs, liver, and spleens relatively sterile (Sierra et al., 2010). The aforementioned findings raise the possibility of developing potential subunit and/or live-attenuated vaccine candidates for *A. hydrophila*, which is an emerging human pathogen.

### *Aeromonas* T2SS cytotoxic enterotoxin (act) and T6SS Hcp effector

In addition to a functional T3SS, *Aeromonas* species also possess a T6SS (Suarez et al., 2008) as well as secrete a potent enterotoxin Act (Table 2) via the T2SS (Chopra and Houston, 1999). In efforts to better understand the host cell response to Act, our laboratory obtained transcriptome profiles of Act-exposed murine RAW 264.7 cells. Not surprisingly, of the 76 differentially expressed genes identified in Act-treated macrophages, many were involved in immune responses, including inflammation (Galindo et al., 2003). Additionally, several apoptosis-related genes were also found to be up-regulated including (but not limited to) Bcl-10 (promotes activation of NF-κB and maturation of pro-caspase 9), BimEL (involved in p38 and JNK-associated apoptosis), and TNF receptor associated factor 1 (TRAF1, which regulates activation of NF-κB and JNK). These transcriptome results with respect to apoptosis-related genes were confirmed by performing real-time PCR as well as functional assays for apoptosis (Galindo et al., 2003).

Since primary host cells may vary in their responses to a stimulant compared to the transformed cell lines, our laboratory evaluated transcriptome profiles of primary murine peritoneal macrophages after treatment with Act. We observed 66% differential gene expression, mirroring our results seen with Act-exposed RAW 264.7 cells (Galindo et al., 2004a,b). However, differential expression of 28 genes unique to primary macrophages was also observed. The pro-apoptotic B-cell leukemia/lymphoma 2 (Bcl-2) and Myeloid differentiation primary response 116 (MyD116) genes were upregulated, while interferon consensus binding proteins 8 (IRF-8—involved in immune responses) was downregulated in Act-treated primary cells (Galindo et al., 2004a). When the effect of Act on human HT-29 colonic epithelial cells' transcriptome profiles was evaluated, we noted upregulation of genes involved in immune responses (e.g., IL-8) and apoptosis (e.g., Bcl-2-like genes) as well as phosphorylation of MAPKs (e.g., p38 kinase, extracellular signal-regulated kinase 1/2 [ERK1/2], and JNK) (Galindo et al., 2005), mirroring what was observed earlier in our mouse macrophage studies (Galindo et al., 2003, 2004a,b). Further, through proteomic analysis, we determined that Act increased phosphorylation/activation of cyclic AMP-response element binding protein (CREB), c-Jun, protein kinase C, and signal transducer and activator of transcription 3 (STAT3) (Galindo et al., 2005).

Realizing that Act induces apoptosis in both cultured and primary macrophages, we elucidated the molecular mechanisms and specifically interrogated the MAPK signaling pathway. We found that, in various cell types, Act exposure resulted in activation of JNK and ERK1/2. Furthermore, Act induced activation/phosphorylation of MAPK upstream factors MKK3/6, MKK4 and MAP/ERK kinase 1 (MEK1) as well as downstream transcription factor c-Jun (Galindo et al., 2004b). With regards to apoptosis, Act induced classical membrane blebbing, increased production of mitochondrial cytochrome *c* and apoptosis-inducing factor, in addition to caspase-3, -8, and -9 activation (Galindo et al., 2004b). When we screened for interactions between Act and both human and yeast proteins (using proArrays), Act was found to bind 9 human proteins (out of ~1800 proteins screened). Of the 9, synaptosomal-associated protein 23 (SNAP23), galectin-3, and guanylate kinase 1 (GUK-1) were knocked down in murine macrophages and HT-29 epithelial cells (using small inhibitory RNA), with the former two resulting in reduced induction of apoptosis following Act exposure (Galindo et al., 2006a,b). Interestingly, we also observed that DNA adenine methyltransferase (Dam) and Glucose inhibited division protein (GidA) both work to positively influence *act* gene expression and its associated hemolytic activity (Erova et al., 2012). Interestingly, the Gram-positive pathogen, *Staphylococcus aureus*, also secretes a potent, pro-apoptotic enterotoxin, the superantigen enterotoxin B. The aforementioned enterotoxin specifically targets T-cells and activates FAS receptor signaling (Ulett and Adderson, 2006 and references therein).

The *Aeromonas* T6SS has two identified effector protein substrates, Hcps and VgrGs (Sha et al., 2013). *A. hydrophila*'s Hcp (Table 2) is a powerful effector substrate and once translocated into the targeted host cell cytoplasm, apoptosis ensues following caspase 3 activation (Suarez et al., 2008). We also demonstrated that Hcp paralyzes macrophages thereby preventing phagocytosis (Suarez et al., 2010). Curiously, multiple copies of Hcp are present in T6SS-possessing bacteria suggesting either redundancy of function and/or dosage-related functional influences (Mougous et al., 2006). The *Aeromonas* gene duplications and various alleles are likely a byproduct of co-evolution occurring in both bacterial pathogens and their respective hosts.

In *A. hydrophila* SSU, the 2 Hcp paralogs cluster to two regions of the chromosome and influence virulence-associated properties differently, demonstrating little functional redundancy (Seshadri et al., 2006; Suarez et al., 2008; Sha et al., 2013). Hcp-2, located inside the T6SS cluster appeared to function structurally in forming the T6SS apparatus while Hcp-1, located at a distal chromosomal site functioned more as an effector (Sha et al., 2013). More specifically, only Hcp-1 worked to negatively regulate bacterial motility and protease production (both required for optimal virulence) whereas both paralogs were required for optimal virulence and dissemination to peripheral organs in a murine model of infection (Sha et al., 2013). When considering the impressive arsenal available to *A. hydrophila* that includes a T3SS, a T6SS as well as a potent T2SS secreted Act, it becomes less surprising that human infections caused by this emerging pathogen are on the rise.

## Conclusion

In context of an intricate host-bacterial pathogen co-evolutionary paradigm, at times it become difficult to determine whether the resulting outcomes better benefit the host or the pathogen. For example, following inhibition of NF-κB and MAPK signaling pathways, *Yersinia* species through their generic PAMP/danger signals (e.g., LPS or flagellin), can induce pyroptosis a specialized inflammation-associated apoptosis that involves the activation of caspase 1 (Philip and Brodsky, 2012 and references therein). Inflammation is, in reality, a double-edged sword. If of short duration and localized, it can serve to reduce extent of bacterial infection preventing systemic spread of the pathogen. However, if persistent and/or systemic, inflammation can damage host tissue and potentially promote bacterial spread contributing to bacterial pathogenesis. These two scenarios underscore the intricacies of the host-pathogen interaction as well as reveal how co-evolution can be shaped. The yersiniae T3SS effector, YopJ, is a perfect example of such an ambiguity. Despite its potent immunomodulatory capabilities, YopJ was largely dispensable for virulence in a rat model of bubonic plague (Lemaitre et al., 2006). Further, there has been evolutionary selective pressure against excessive YopJ secretion in order to achieve maximal virulence during plague infections (Zauberman et al., 2006; Brodsky and Medzhitov, 2008). What does this all mean? Does YopJ's powerful ability to induce inflammation benefit the pathogen or the host? Perhaps viewed in this light, the cost to benefit ratio nears “1” making YopJ a “circumstantial virulence factor” depending on the *Yersinia* pathogen in question, the route of infection, the immunodisposition of the host, etc.

The T3SS is a powerful vehicle of effector protein delivery shared by many Gram-negative pathogens. The pathogenic *Aeromonas* species also possess a functional T3SS that delivers 4 effector proteins into targeted host cells. The YopJ homolog, AopP disrupts NF-κB signaling downstream of IKKB but, unlike YopJ in the yersinaie, does not disrupt the MAPK signaling pathway (Fehr et al., 2006). AexU, is an extremely versatile *Aeromonas* T3SS bifunctional effector that possesses both GAP activity (like the yersiniae YopE) as well as ADP-RT activity. Like YopJ, AexU induces apoptosis and targets NF-κB signaling. However, unlike in the yersinaie which lack clearly defined T6SS-virulence factors, *Aeromonas* species possess well-defined T6SS-associated virulence factors and even a T2SS-secreted toxin (Act) creating a much wider arsenal. The T6SS Hcp paralogs sharing limited functional redundancy suggest that co-evolution might have shaped the gene duplication event as well as provide the necessary selection pressure that maintains the multiple copies in the chromosome. Taken together, T3SSs effectors in both the yersiniae and *Aeromonas* species as well as T2- and T6SS effectors in *Aeromonas* species converge on modulating the host immune response to promote bacterial virulence.

### Conflict of interest statement

The authors declare that the research was conducted in the absence of any commercial or financial relationships that could be construed as a potential conflict of interest.
